# Comparative Effects of Two High-Intensity Intermittent Training Programs on Sub-Elite Male Basketball Referees’ Fitness Levels

**DOI:** 10.3390/sports12020051

**Published:** 2024-02-02

**Authors:** David Suárez-Iglesias, Alejandro Rodríguez-Fernández, Alejandro Vaquera, José Gerardo Villa-Vicente, Jose A. Rodríguez-Marroyo

**Affiliations:** 1 VALFIS Research Group, Department of Physical Education and Sports, Institute of Biomedicine (IBIOMED), Universidad de León, 24071 León, Spain; dsuai@unileon.es (D.S.-I.); alrof@unileon.es (A.R.-F.); avaqj@unileon.es (A.V.); jg.villa@unileon.es (J.G.V.-V.); 2 Faculty of Physical Activity and Sports Sciences, Universidad de León, 24071 León, Spain

**Keywords:** repeated sprints, RSA, HIIT, training, performance, team sports

## Abstract

This study aimed to compare the effects of an 8-week short-term training program, comprising repeated sprints or running-based high-intensity intermittent training (HIIT), on the aerobic fitness and repeated sprint ability (RSA) performance of sub-elite basketball referees. Twenty male referees participated in supervised training sessions twice a week. They were randomly assigned to either the RSA-based group (RSAG) or the running-based HIIT group (HIITG). The RSAG conducted 3–4 sets of 8 × 20-m all-out sprints, while the HIITG performed 2–3 sets of 6 × 20-s runs at 90% of their maximal velocity achieved in the 30–15 intermittent fitness test (30–15_IFT_). Referees underwent a graded exercise test on a treadmill, the 30–15_IFT_, and an RSA test before and after the training program. Both groups showed significant improvement (~3%) in the fastest (22.6 ± 1.4 vs. 23.4 ± 1.7 and 22.0 ±1.9 vs. 22.4 ± 1.7 km·h^−1^ in RSAG and HIITG, respectively) and mean (21.5 ± 1.2 vs. 22.4 ± 1.4 and 21.3 ± 1.8 vs. 21.7 ± 1.6 km·h^−1^ in RSAG and HIITG, respectively) sprint velocity of the RSA test (*p* < 0.05). Moreover, positive changes (*p* < 0.05) were observed in the 30–15_IFT_ maximal velocity (18.6 ± 1.1 vs. 19.3 ± 1.0 and 19.4 ± 0.9 vs. 20.5 ± 0.9 km·h^−1^ in RSAG and HIITG, respectively). In conclusion, an 8-week training intervention using either RSA or running-based HIIT led to similar improvements in referees’ RSA performance and specific aerobic fitness measures. These findings could assist in devising tailored training programs for basketball referees.

## 1. Introduction

The physical demands of basketball matches have been extensively documented for players across various competitive levels [1]. Being an intermittent sport, basketball involves frequent bursts of high-intensity, short-duration activities such as jumping, sprinting, and accelerating, interspersed with periods of lower to moderate intensity actions (e.g., standing, walking, and jogging) [2]. Despite the pivotal role referees play in basketball competitions, there has been relatively less research into their physiological and physical demands. Referees endure substantial exercise volume and intensity throughout matches and the competitive season [3]. Officiating official matches constitutes intermittent moderate-intensity activity [4], wherein referees sustain an exercise intensity between 60–85% of their maximum heart rate (HR_max_) [5,6]. Estimated metabolic demands hover around 5 METs, emphasizing the significant reliance on the aerobic energy pathway [7]. Basketball referees cover distances ranging from 4–6 km per match, primarily involving walking, standing, jogging, or executing short lateral movements, punctuated by brief instances of intense activity like running or sprinting [8,9].

Creating and implementing appropriate training programs for basketball referees is essential to effectively meet the demands of competition [10]. However, several barriers hinder referees from attaining and sustaining optimal physical fitness. For instance, many referees, including those at the international level, lack tailored fitness regimens due to their tightly packed schedules [11]. Even when accessible online, such as through manuals or video tutorials, specific workouts and structured training plans lack oversight [12]. Referees themselves bear the responsibility of managing their training, potentially impacting their commitment to meeting physical fitness standards and adhering to a structured weekly regimen [13].

Thus far, insufficient attention has been given to designing optimal training programs aimed at enhancing referees’ physical fitness [3,4]. Specifically, training sessions should prioritize the development of acceleration, speed, and aerobic capacity both pre-season and throughout the competitive period [3,8,14]. Implementing high-intensity intermittent training (HIIT) stands as a promising, time-efficient approach to bolster referees’ physical fitness [15]. For instance, prescribing HIIT using a 1:12 work-to-rest ratio could notably enhance basketball referees’ intermittent high-intensity endurance [4]. Conversely, some researchers [16] advocate for repeated-sprint training, such as 2 × 15-m shuttle sprints with a passive recovery of 20–25 s between repetitions, due to its positive correlation with on-field referee performance. Additionally, a training protocol involving 20–30-m sprints with directional changes and 20–30 s recovery intervals may significantly enhance referees’ repeated-sprint performance during matches [4].

To date, only one study has endeavored to elucidate the impact of a training regimen on the physical fitness of basketball referees. This study employed a traditional training program encompassing various activities (i.e., 20-m progressive runs, sprint training, strength-based circuit training, alternating pace runs of 5–10 min, and 30-min continuous endurance running), which notably enhanced the repeated sprint ability (RSA) and anthropometric characteristics of sub-elite referees [17]. However, this program, conducted over five weeks, demanded a significant time commitment (averaging about 75-min per session, thrice weekly). Such a requirement poses a challenge for this demographic, often balancing full-time professions alongside sports commitments [18], further complicated by geographical limitations, work constraints, and official duties that disrupt regular training schedules [15].

In light of these challenges, there arises speculation that employing shorter, more specific training sessions (focusing on RSA-based or running-based HIIT formats) could present an alternative strategy to develop the desired physical attributes crucial for basketball referees. Therefore, this study aimed to explore whether an eight-week training program, involving either repeated sprints or running-based HIIT, conducted twice weekly, could generate positive improvements in critical aspects of the physical fitness of sub-elite male basketball referees, such as aerobic fitness and RSA performance.

## 2. Materials and Methods

### 2.1. Design

A randomized, longitudinal, open-label experimental design was employed. Pre-tests and post-tests were conducted two weeks before and after an 8-week training intervention. All testing sessions were conducted consistently at a fixed time (18:00–19:00 h) and in comparable environmental conditions (20–25 °C, 30–35% relative humidity, and an altitude of 837 m).

### 2.2. Participants

Twenty male volunteers from the Regional Spanish Basketball Referees Association participated in this study (Table 1). They served as active basketball referees at the sub-elite (state/regional) level [14], regularly training twice per week and officiating at least once weekly during the regular season. According to The Physical Activity Readiness Questionnaire for Everyone (PAR-Q) [19], none of the participants reported any health or lifestyle issues prior to commencing the study. Individuals were randomly assigned to either the RSA-based group (RSAG; n = 9) or the running-based HIIT group (HIITG; n = 11) using block randomization in a one-to-one ratio allocation [20]. The total sample size was a priori (n = 20) calculated using the software G*Power v. 3.1.9.6 [21]. The α value was stablished at 0.05 with a power of 0.80. Furthermore, taking into consideration insights from previous studies [22,23,24,25,26,27,28,29], it was anticipated that the effect sizes would range from moderate to large. Therefore, a Cohen’s *f* value of 0.35 was used for the analysis.

Throughout the study period, the referees were encouraged to maintain their normal dietary patterns and refrain from engaging in any additional physical activities beyond their usual routines. Written informed consent was obtained before the study commenced. The protocol was approved by the University of León Ethical Committee (Spain) (ETICA-ULE-017-2019) and adhered to the principles outlined in the Declaration of Helsinki.

### 2.3. Procedures

#### 2.3.1. Training Intervention

Throughout the 8-weeks training intervention, participants engaged in supervised training sessions twice a week (Tuesdays and Thursdays) and officiated in basketball matches once per week (Saturdays). Each training sessions included a standardized warm-up and cool-down routine. The warm-up (~15 min) included low-intensity running followed by joint mobility exercises (e.g., shoulder rotations, hip and ankle movements), agility drills, dynamic stretching exercises and a series of 4 progressive 30-m sprints. The cool-down (~10 min) involved low-intensity running and static stretching exercises. Stretching exercises (adductors, quadriceps, hamstrings, calves and Achilles) were held for 15-s and repeated four times with a 15-s rest between each repetition. All stretches were performed below the maximal point of discomfort, avoiding any sensation of pain.

However, the main part of the training session varied based on the training program followed by the referees. RSAG’s training involved the repetition of 20-m all-out sprints. Specifically, 8 repetitions were performed, interspersed with 30-s of active recovery, during which the referees jogged back to the starting line. The training volume increased over the intervention period, with 3 series in week 1, 4 series in weeks 2–5 and 5 series in weeks 6–8. The recovery between series was passive and lasted 2 min. The total training time for this specific RSAG segment ranged from 16 to 28 min. In contrast, the HIITG followed a HIIT regimen involving short intervals of 20-s runs performed at 90% of their velocity at the end of the 30–15 Intermittent Fitness Test (30–15_IFT_), incorporating directional changes at varying angles. The running pace was indicated through auditory signals. During week 1, referees performed 2 series of 6 repetitions, increasing to 3 and 4 series in weeks 2–5 and 6–8, respectively. In this training group, a 20-s passive recovery and a 5-min rest were performed between repetitions and series, respectively. The specific training time for this segment ranged from 13 to 31 min.

To quantify the exercise intensity during the specific training, referees provided their rating of perceived exertion (RPE) immediately after completing this period using a CR-10 scale [30]. Finally, both groups concluded with the same cool-down period involving low-intensity running and stretching exercises.

#### 2.3.2. Testing Sessions

Before and after the training program, referees underwent three separate testing sessions, separated by at least 48 h over two weeks. The initial testing session involved anthropometric measurements and a maximal aerobic capacity test. In the subsequent two sessions, referees performed an RSA test and a 30–15_IFT_, respectively, on a synthetic indoor basketball court. Verbal encouragement was given to motivate participants to exert maximum effort. Prior to each testing session, individuals received familiarization with the procedures to mitigate any potential learning effect.

All tests commenced after a standardized 15-min warm-up comprising submaximal running and 5-min of free stretching. Referees completed the tests wearing their usual running shoes. In the 24 h preceding the testing sessions, referees were instructed to avoid vigorous exercise and adhere to a carbohydrate-rich diet. Additionally, they were asked to abstain from consuming caffeine within 6 h before testing.

##### Anthropometric and Aerobic Fitness Assessment

Height (seca 213, seca GmbH & Co. KG, Hamburg, Germany) and body mass (Tanita MC-780U, Tanita Corp., Tokyo, Japan) were measured upon individuals’ arrival at the laboratory. Additionally, estimated body fat percentage were determined employing bioelectrical impedance principles with a multi-frequency segmental body composition analyzer (Tanita MC-780U, Tanita Corp., Tokyo, Japan).

Subsequently, participants underwent a graded exercise test on a treadmill (h/p/cosmos pulsar, Cosmos Sports & Medical GMBH, Nussdorf-Traunstein, Germany). The test commenced at 6 km·h^−1^, with the velocity increasing by 1 km·h^−1^ every minute until voluntary exhaustion. The treadmill grade remained at 1% throughout the test. Continuous respiratory gas exchange was recorded using a breath-by-breath system (Medisoft Ergocard, Medisoft Group, Sorinnes, Belgium), calibrated per the manufacturer’s guidelines. Heart rate (HR) response was also monitored via a 12-lead electrocardiogram (Medisoft Medcard, Medisoft Group, Sorinnes, Belgium). VO_2max_ was recorded as the highest values obtained within the 30-s preceding exhaustion. Maximal velocity (vGXT) was determined as the highest velocity participants could sustain for a complete stage plus the interpolated velocity from incomplete stages [31].

Confirmation of maximal effort required meeting at least two of the following criteria [32]: a VO_2_ plateau (≤150 mL·min^−1^), respiratory exchange ratio ≥1.15, and heart rate within ±10 beats of the theoretical maximal heart rate. The ventilatory (VT) and respiratory compensation thresholds (RCT) were identified based on specific criteria [33]: an increase in both ventilatory equivalent for oxygen (VE·VO_2_^−1^) and end-tidal oxygen pressure (PETO_2_) with no concomitant increase in the ventilatory equivalent for carbon dioxide (VE·VCO_2_^−1^) for VT, and an increase in both VE·VO_2_^−1^ and VE·VCO_2_^−1^ and a decrease in the end-tidal carbon dioxide pressure for RCT.

##### Repeated Sprint Ability Test

After the standardized warm-up, the referees completed three 20-m sprints, a distance that has been recommended for assessing RSA in basketball referees [17]. The best time recorded among these sprints was considered the criterion score [34]. Following a 5-min recovery period, the RSA test was conducted, comprising eight maximal 20-m sprints with 25-s of active recovery (running back to the starting line and waiting for the next sprint) in between. Two seconds before the commencement of each sprint, participants assumed a static standing position, placing their front foot 50-cm behind the first photocell gate (DSD Laser System, DSD Inc., León, Spain) set at a height of 50-cm [22].

Results from the RSA test were only considered valid when the time of the first sprint was ≤2.5% slower than the criterion score [35]. If this condition was not met, the test was terminated immediately, and individuals were asked to repeat the RSA test after a 5-min rest [35]. The fastest (RSA_best_) and mean (RSA_mean_) sprint times were determined [34]. To quantify fatigue during the test, the percentage decrement score (Sdec) was calculated using the formula: Sdec = (Sum of all RSA sprint times/(RSA_best_ × total number of sprints) × 100) − 100 [36].

##### The 30–15 Intermittent Fitness Test

The test took place on an indoor basketball court. The 30–15_IFT_ consisted of 30-s shuttle runs, requiring participants to shuttle back and forth between two lines placed 28-m apart. These running intervals were alternated with 15-s passive recovery periods, conducted at a walking speed [37]. The initial run began at 8 km·h^−1^, and the velocity increased by 0.5 km·h^−1^ with each subsequent 30-s stage. Running speeds were dictated using a pre-recorded audio file. Referees were instructed to complete as many stages as possible. The test concluded either upon voluntary exhaustion or when participants could no longer sustain the required running velocity for three consecutive intervals. The velocity achieved during the final completed stage was recorded (V_IFT_).

### 2.4. Statistical Analyses

The results were expressed as mean ± standard deviation (*SD*). The assumption of normality was verified using the Shapiro-Wilk test. A repeated-measures 2-way ANOVA (intervention [RSAG vs. HIITG]) × (time [pre-training vs. post-training]) was used to assess the effects of the training programs on referees’ physical fitness. A one-way ANCOVA was used to establish differences between groups’ relative changes in performance, using the pre-training values as covariates.When a significant *F* value was found, Bonferroni’s post hoc test was applied to establish significant differences between means. Finally, an unpaired *t*-test was employed to analyze the differences between the exercise demands of RSAG and HIITG. Statistical significance was set at *p* < 0.05. Cohen’s *d* was calculated as an indicator of effect size (ES)*,* values of <0.20, 0.20 to 0.50, 0.51 to 0.80, and >0.80 were rated as trivial, small, moderate, and large effects, respectively. We obtained meaningful changes (0.2 × between subjects *SD*) and typical error of measurement (TE) to determine the effectiveness of training programs (impairment, trivial, or improvement) [38]. Likely, limits for the true value were calculated (observed changed ± TE), and the intervention was classified as beneficial or harmful when they lied beyond the meaningful changes [38]. Data analyses was performed using IBM SPSS for Windows v25 (SPSS Inc, Chicago, IL, USA).

## 3. Results

**Figure 1:**
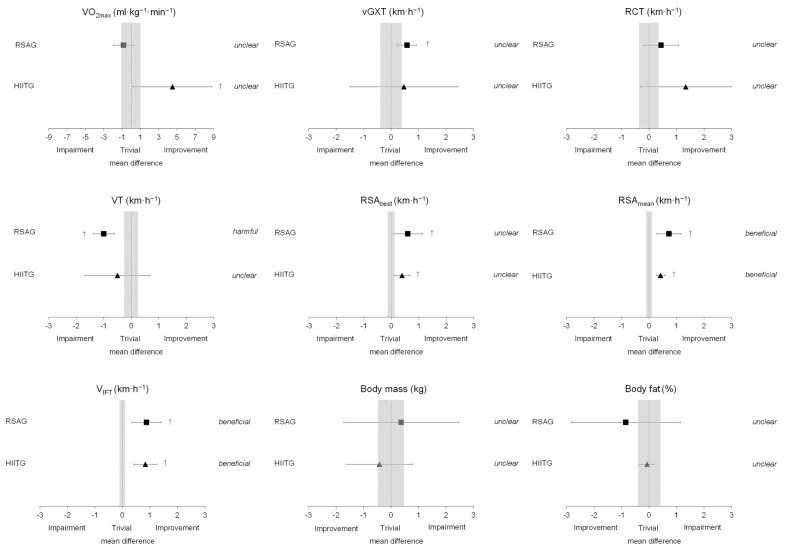
Mean difference for repeated-sprint ability training group (RSAG) and running-based high-intensity intermittent training group (HIITG). Values are mean ± TE. Trivial areas were computed from the meaningful changes. †, mean difference≥ or ≤meaningful change and TE.

Referees’ body mass and body fat percentage showed no significant differences between groups (Table 1). The time factor, type of intervention, and their interaction did not have any effect on body mass and body fat. The changes observed in these variables were rated as unclear (Figure 1).

Although overall aerobic performance showed no significant differences between groups at pre- and post-training (Table 2), a greater change (*p* < 0.05) in VO_2max_ was specifically noted in the HIITG following the training program. Moreover, large improvements in RCT velocity were observed in this training group. Notably, although the intervention resulted in reductions in VT for both groups, statistically significant differences (*p* < 0.05) were only observed in RSAG. On the contrary, the specific endurance (i.e., V_IFT_) of the referees significantly improved (*p* < 0.05) after training in both groups (Table 2). Assessing the significant changes and potential boundaries of true values, both training groups yielded positive adaptations in V_IFT_ (Figure 1). However, a detrimental decline in VT velocity was observed specifically in RSAG.

**Figure 2:**
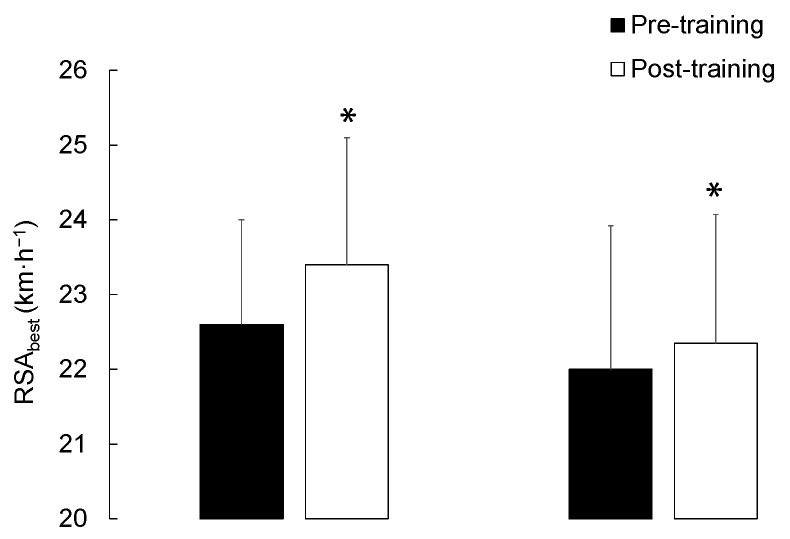

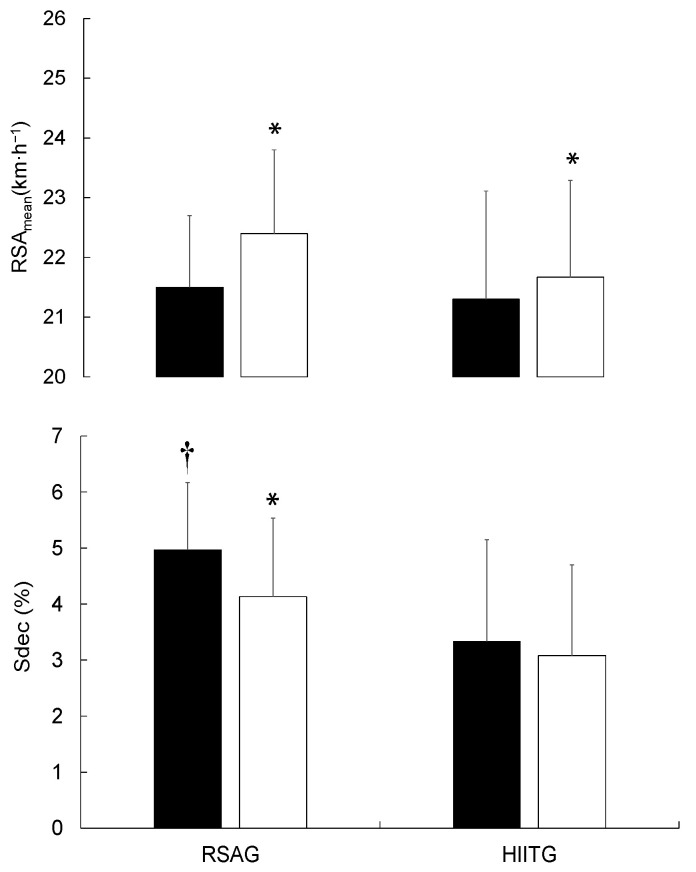
Fastest speed (RSA_best_), mean speed (RSA_mean_), and percentage of decrement (Sdec) of the repeated sprint ability test according to the intervention group. *, significant difference between Post- and Pre-training intervention (*p* < 0.05). †, significant difference between RSAG and HIITG (*p* < 0.05).

Both training programs significantly (*p* <0.05) improved the RSA_best_ values (*d* = 0.45 and 0.16 in RSAG and HIITG, respectively) as well as the RSA_mean_ values (*d* = 0.69 and 0.23 in RSAG and HIITG, respectively) (Figure 2). Figure 1 presents an analysis of the beneficial changes observed in both groups. Specifically, the observed percentage changes for RSA_best_, RSA_mean_, and Sdec in RSAG and HIITG were 3.4 ± 2.8%, 4.2 ± 2.6%, −0.8 ± 0.8% and 1.8 ± 1.0%, 1.9 ± 1.1%, −0.3 ± 1.2%, respectively.

Finally, the daily training volume was similar between groups (51.4 ± 5.2 and 53.2 ± 5.9 min in RSAG and HIITG, respectively; *d* = 0.32). However, following the intervention conditioning exercises, the mean perceived exertion (RPE) was notably higher (*p* < 0.05, *d* = 0.80) in HIITG (7.1 ± 0.9) compared to RSAG (6.2 ± 1.3).

## 4. Discussion

This open-label study represents the first attempt to implement and compare two distinct training methodologies (RSA-based vs. running-based HIIT training programs) while evaluating their impact on the physical fitness of sub-elite male basketball referees. Our results indicated aerobic adaptations in both training groups, with significant improvements observed in V_IFT_ for both RSAG and HIITG as shown in Table 2. Beneficial differences in both training groups after the intervention period were analyzed (Figure 1). Furthermore, the observed magnitude of change in both VO_2max_ and RCT velocity was substantial following the training intervention in the HIITG. Conversely, significant improvements in RSA performance (i.e., RSA_best_ and RSA_mean_) were comparable in both groups (Figure 1 and Figure 2).

The RSA-based and running-based HIIT training programs both featured repetitions of short running bouts interspersed with brief recovery periods [39,40]. An 8-week intervention with two training sessions per week appeared to enhance specific aerobic fitness parameters in our sample of sub-elite male basketball referees (V_IFT_). This finding aligns with previous research in team sports [23,24,25], which has shown that similar HIIT protocols effectively improve aerobic performance. Notably, previous studies reported enhancements such as a 23% increase in the total distance covered during the YoYo IR1 in handball players [24], a 3.5% improvement in V_IFT_ among basketball players [25], and a 5% increase in soccer players [23].

It’s essential to highlight our use of VO_2max_ and ventilatory thresholds to evaluate the referees’ aerobic performance. Our findings indicated that following the intervention, RSA-based training had a minimal effect on VO_2max_ (Table 2), contrary to previous observations suggesting that sprint interval training could moderately enhance aerobic capacity (~8%), with an average improvement of 3.6 mL·kg^−1^·min^−1^ in VO_2max_ (from ~45 to ~49 mL·kg^−1^·min^−1^) [41]. These results were observed in a specific population of young, healthy, predominantly sedentary, and recreational subjects. Thus, it is plausible to consider that a ceiling effect within our study might have limited the magnitude of changes in VO_2max_. Our participants exhibited values around 18% higher than those reported in prior studies [41], and even 4% higher than those previously measured in male basketball referees [42].

The absence of improvement in VO_2max_ post-training does not necessarily imply no adaptations from our intervention. Other parameters, such as peak cardiac output and mitochondrial oxidative capacity, might have been enhanced without a corresponding change in VO_2max_ [43]. Indeed, RSA-based training resulted in a significant improvement in maximal treadmill velocity during the graded exercise test (~4%, moderate effect size). Several explanations might account for this combination of results. Repeated sprint-based training might have induced more peripheral adaptations than central ones [44]. Peak velocity achieved in the treadmill test demonstrates a stronger association with running performance than VO_2max_, encompassing the locomotor aspect of maximal aerobic function and carrying a significant neuromuscular influence [45].

An unexpected finding was the significant decrease (9.7%, large effect size) in running speed at VT during the graded exercise test for RSAG, contrasted with a 4.6% decline (moderate effect size) in HIITG. Several potential explanations can be considered. Previous literature suggests that an improvement in RSA_mean_ without concurrent changes in Sdec may signify anaerobic benefits, even in the absence of alterations in aerobic performance [22,26]. Furthermore, studies have indicated that training programs incorporating short-duration efforts (<10-s sprints) prompt increased activity in anaerobic enzymes, a rise in the proportion of type II muscle fibers [27,46], and an enhanced sarcoplasmic reticulum Ca^2+^ release rate [47]. These outcomes bear implications for effective training interventions tailored for basketball referees. Considering this aspect, an inclusive training approach that caters to their specific requirements may involve integrating low-intensity running-based exercises alongside high-intensity conditioning stimuli. This strategy aims to enhance or sustain their performance at an intensity corresponding to VT.

Both training interventions resulted in improved RSA_mean_, aligning with earlier findings in sub-elite male basketball referees by Bayon et al. [17], who conducted five weeks of specific physical training. Following their intervention, these authors noted a 5.3% increase in RSA_mean_ for the experimental group, reporting significant differences favoring the experimental group over the control. Indeed, this parameter holds significance as a valid indicator of RSA performance [22,48]. This discovery bears implications for designing referees’ training programs, as repeated sprinting stands as a crucial physical ability in basketball referees due to the unique demands of their match activities [3,16].

The enhancement observed in the HIITG might be attributed to the efficiency of HIIT in improving the ability to recover between sprints [49]. However, this improvement might not be associated with a better post-training Sdec, as observed in our study. Similar findings were reported previously among elite male junior basketball players. Delextrat et al. [25] noted significant improvements in RSA after a six-week HIIT intervention (intermittent running at 95% of V_IFT_ for 15-s followed by 15-s of active recovery). However, they observed a smaller Sdec post-training, contrasting with our results. This disparity could be linked to differences in the protocols used to assess RSA. While we employed a test comprising 8 repetitions of 20-m, Delextrat et al. [25] utilized two repetitions of 15-s all-out sprints. Despite Sdec being associated with high-intensity activities such as running and sprinting in male basketball referees [16], it seems that this variable might not be a reliable index of RSA performance in team sport athletes [50]. It’s worth noting the potential influence of initial sprint performance on Sdec [51]. Another plausible explanation could be the training intensity in our intervention. Studies in handball players demonstrated that exercise intensities ranging from 100–130% of maximal aerobic velocity (10–20 s with 10–20 s of recovery) performed twice weekly during a 7-week in-season program significantly improved RSA performance (~3% in RSA_best_ and RSA_mean_, and ~10% in Sdec) [28]. In our study, HIITG similarly improved RSA_best_ and RSA_mean_ by approximately 2%; however, the increase in Sdec was notably lower (~0.5%).

After the intervention, there was a significant improvement (~3.0%) in both RSA_mean_ and RSA_best_ for RSAG, while no significant changes were detected for Sdec (~1.0%). The effects of repeated sprint training on RSA performance appear controversial in literature. Some studies with team sport athletes have demonstrated improvements [52], while others have reported no changes [53]. Additionally, a meta-analysis of controlled and non-controlled trials suggested a potentially moderate beneficial effect of repeated sprint training on RSA [29]. However, the observed improvement in RSA_mean_ without a subsequent change in Sdec in our investigation might indicate an enhancement in referees’ anaerobic performance [54], rather than in their ability to recover between sprints [26].

Finally, regarding anthropometric measurements (i.e., body mass and body fat percentage), we did not observe apparent effects of training on our sample. These findings align with other research involving sub-elite male basketball referees [17], which found no significant differences in anthropometric metrics following a 5-week specific training intervention. However, it’s crucial to interpret these results cautiously as we did not control dietary intake, which could impact body mass or body fat percentage.

One strength of this study lies in the comprehensive assessment methods employed, combining laboratory-based and field-based fitness tests. Additionally, to ensure consistency, all participants trained together in groups and underwent field-based tests in the same venue, guided by the same qualified personnel. Moreover, the average duration of specific activities in this intervention was less than 23-min per training session, representing a reduction compared to a previous training program [17]. This aspect could be beneficial for referees with limited exercise time seeking improvements in RSA performance. However, our study has potential limitations. It was an open-label study with a small sample size and a short intervention, potentially influencing the results and limiting generalization. Another limitation was the absence of a non-active control group, hindering the assessment of non-training factors that might impact the dependent variables. Lastly, considering all participants were sub-elite male basketball referees, caution should be exercised, and further studies are necessary to validate whether these findings can be extrapolated to referees of different expertise levels, ages, and female referees.

## 5. Conclusions

An 8-week training intervention, involving either repeated-sprint training or running-based HIIT, resulted in comparable enhancements in the referees’ RSA performance. Additionally, both training programs resulted in improvements in specific aerobic fitness. Nonetheless, it’s noteworthy that a decrease in running speed at VT during the graded exercise for both training groups was observed, highlighting the necessity for an integrated training approach that addresses low-intensity exercise stimuli.

These findings could potentially aid in devising tailored training programs for sub-elite male basketball referees with limited time for physical training. However, given the small sample size, caution is warranted as these findings may not be generalizable to other male basketball referees across different categories and physical fitness levels. Consequently, further studies with larger sample sizes are imperative to validate these results.

## Figures and Tables

**Table 1:** Basketball referees’ anthropometric characteristics (mean ± *SD*).

	Group	Pre-Training	Post-Training	Δ (%)	Cohen *d* (Rating)
Refereeing experience (year)	RSAG	8.2 ± 5.7	N/A		
	HIITG	8.5 ± 6.6	N/A		
Age (year)	RSAG	25.6 ± 5.8	N/A		
	HIITG	25.5 ± 9.7	N/A		
Height (cm)	RSAG	177.2 ± 0.1	N/A		
	HIITG	182.5 ± 0.3	N/A		
Body mass (kg)	RSAG	72.7 ± 8.0	71.7 ± 7.3	−1.1 ± 3.0	0.13 (trivial)
	HIITG	76.1 ± 6.3	75.4 ± 5.4	−0.8 ± 1.9	0.12 (trivial)
Body fat (%)	RSAG	15.3 ± 4.0	14.6 ± 5.6	−0.7 ± 1.6	0.17 (trivial)
	HIITG	13.4 ± 5.9	13.3 ± 5.7	−0.1 ± 0.4	0.02 (trivial)

RSAG, repeated-sprint ability training group; HIITG, running-based high-intensity intermittent training group. N/A, not applicable.

**Table 2:** General and specific aerobic performance in RSA-based (RSAG) and running-based HIIT (HIITG) training groups (mean ± *SD*).

	Group	Pre-Training	Post-Training	Δ (%)	Cohen *d* (Rating)
VO_2max_ (ml·kg^−1^·min^−1^)	RSAG	55.2 ± 7.2	54.5 ± 6.5	−1.1 ± 2.1	0.10 (trivial)
	HIITG	53.7 ± 4.9	56.0 ± 5.3	4.4 ± 7.0 †	0.45 (small)
vGXT (km·h^−1^)	RSAG	16.1 ± 1.2	16.8 ± 1.3	4.5 ± 2.7	0.56 (moderate)
	HIITG	16.9 ± 1.7	17.1 ± 1.1	1.8 ± 10.5	0.14 (trivial)
RCT (km·h^−1^)	RSAG	13.6 ± 1.2	14.3 ± 1.2	5.6 ± 6.1	0.58 (moderate)
	HIITG	13.3 ± 1.3	14.6 ± 1.4	9.8 ± 8.9	0.96 (large)
VT (km·h^−1^)	RSAG	10.7 ± 0.9	9.6 ± 0.5 *	−9.7 ± 4.1	1.51 (large)
	HIITG	10.9 ± 0.9	10.4 ± 0.8	−4.6 ± 12.8	0.59 (moderate)
V_IFT_ (km·h^−1^)	RSAG	18.6 ± 1.1	19.3 ± 1.0 *	4.2 ± 3.7	0.67 (moderate)
	HIITG	19.4 ± 0.9	20.5 ± 0.9 *	5.4 ± 2.3	1.22 (large)

vGXT, maximal velocity in the graded exercise test; RCT, respiratory compensation threshold; VT, ventilatory threshold; V_IFT_, velocity at the last completed stage in 30–15 IFT. *, significant difference between Post- and Pre-training intervention (*p* < 0.05). †, significant difference between RSAG and HIITG (*p* < 0.05).

## Data Availability

The data that support the findings of this study are available from the corresponding author, upon reasonable request.
